# Racial Disparities in Survival Among Non-Hodgkin Lymphoma Patients: An Analysis of the SEER Database (2007-2015)

**DOI:** 10.7759/cureus.25867

**Published:** 2022-06-12

**Authors:** Faith O Abodunrin, Oluwasegun A Akinyemi, Ademola S Ojo, Kindha Elleissy Nasef, Thomas Haupt, Ayobami Oduwole, Oni Olanrewaju, Bolarinwa Akinwumi, Mary Fakorede, Oluwaseun Ogunbona

**Affiliations:** 1 Internal Medicine, Creighton University School of Medicine, Omaha, USA; 2 Health Policy and Management, University of Maryland School of Public Health, College Park, USA; 3 Surgery, Howard University, Washington DC, USA; 4 Internal Medicine, Howard University Hospital, Washington DC, USA; 5 Obstetrics and Gynecology, Howard University College of Medicine, Washington DC, USA; 6 Internal Medicine, Howard University College of Medicine, Washington DC, USA; 7 Hospital Medicine, The Medina Clinic, Grandview, USA; 8 Pathology, Howard University College of Medicine, Washington DC, USA; 9 Health Sciences and Social Work, Western Illinois University, Macomb, USA; 10 Family Medicine, Howard University College of Medicine, Washington DC, USA; 11 Psychiatry, Ladoke Akintola University, Ogbomoso, NGA; 12 Pathology, Houston Methodist Hospital, Houston, USA

**Keywords:** overall survival (os), minority health, healthcare outcomes, racial disparity, non-hodgkin lymphoma (nhl)

## Abstract

Introduction

Although disparities in cancer survival exist across different races/ethnicity, the underlying factors are not fully understood.

Aim

To identify the interaction between race/ethnicity and insurance type and how this influences survival among non-Hodgkins lymphoma (NHL) patients.

Methods

We utilized the SEER (Surveillance, Epidemiology, and End Results) Registry to identify patients with a primary diagnosis of NHL from 2007 to 2015. Our primary outcome of interest was the hazard of death following a diagnosis of NHL. In addition, we utilized the Cox regression model to explore the interaction between race and insurance type and how this influences survival among NHL patients.

Results

There were 44,609 patients with NHL who fulfilled the study criteria. The mean age at diagnosis was 50.9 ± 10.8 years, with a mean survival of 49.8± 34.5 months. Among these patients, 64.8% were non-Hispanic Whites, 16% were Hispanics, and 10.8% were Blacks. In addition, 76.5% of the study population had private insurance, 16.6% had public insurance, and 6.9% were uninsured. Blacks had the worst survival (HR=1.66; 95% = 1.55-1.78). Patients on private insurance had better survival compared to those with public insurance (HR=2.11; 95% CI=2.00-2.24)

Conclusion

The racial and socioeconomic disparity in survival outcomes among patients with NHL persisted despite controlling for treatment modalities, age, and disease stage.

## Introduction

Non-Hodgkin lymphoma (NHL) is the most common hematopoietic malignancy worldwide, comprising 2.8% of worldwide cancer diagnoses [1]. According to the latest Global Cancer Incidence, Mortality, and Prevalence (GLOBOCAN) data, an estimated 259,793 global deaths were attributable to NHL in 2020, ultimately accounting for 2.6% of all oncological mortality worldwide [1]. In the United States, NHL is the seventh most prevalent cancer and has the sixth-highest mortality among malignancies [2]. In addition, the incidence of NHL has increased by approximately 168% since 1975. Overall, NHL is more prevalent among men, people over 65 years old, and those with autoimmune diseases and a family history of hematological malignancies [3].

NHL is a unique cancer diagnosis comprising over 60 lymphoid malignancies of different molecular origins, with differing clinical manifestations and prognoses ranging from indolent to highly aggressive [4]. NHLs are categorized according to cell type, location, nodal or extranodal, and tumor grade [5]. B-cell lymphomas make up approximately 85% of all NHLs, and T-cell lymphomas make up the remaining 15% [5]. Moreover, each class incorporates precursor and mature neoplasms, subdivided into specific lymphomas [4].

The survival of patients with NHL has improved over the last few decades due to innovations in therapeutics. Newer treatments targeting molecular pathways of specific malignancies have led to partial or complete recovery [3]. For example, the development and implementation of new cytotoxic regimens, stem cell transplant techniques, anti-CD-20 antibodies, and targeted therapies like BCL-2 inhibitors and PD-1 inhibitors have significantly improved outcomes across relapsed and refractory diseases [6].

The five-year survival of NHL in the U.S. has improved from the earliest reported survival rate in 1975 of 46% to a five-year survival of 72.7% between 2010 to 2016, a remarkable improvement of 158% [3]. The current five-year survival for stage I disease at diagnosis is 83.5% while the survival for stage IV disease is 63.3% [3].

However, inequalities in access to advanced treatments and clinical trial participants exist among different racial groups and socioeconomic classes [2]. Recently, studies have identified that racial disparities existed in access to high-quality care, cancer diagnostic services, and service delivery [7]. Moreover, studies show that socioeconomic status and lack of sufficient health insurance coverage were significantly associated with worse survival in lymphoma patients [8].

Though it is evident that the survival of the NHL has improved over the past several decades, improvement may not be equal among all patients, and more research needs to examine disparities between racial groups and socioeconomic classes. The present study aims to identify the interaction between race/ethnicities and insurance type and examine how this influences survival among NHL patients between 18 and 65 years of age.

## Materials and methods

Methods

We collated the data for this study from the Surveillance, Epidemiology, and End Results (SEER) Registry from 2007 to 2015. The SEER database is an authoritative resource for cancer data in the United States. Besides being a reliable source of information on the occurrence and survival of cancer in the U.S, the SEER database is the sole complete and routine supply of population-based information that contains the demography of the patient, the primary site of the tumor, the morphology of the tumor, the cancer stage at diagnosis time, the treatment course, and patient follow-up as well as survival. Moreover, the SEER program is updated yearly and utilized by multitudes of clinicians, researchers, legislators, public health administrators, policymakers, and community groups to investigate the burden of cancer among the American populace. In addition, through the collaborative efforts of national data standards and various national committees, the SEER Program provides high-quality, and error-proofed data with the aid of extensive field edits that correct and avoid errors by checking for missed data as well as verifying codes.

Study population

We studied patients with a primary diagnosis of NHL in the SEER database between January 2007 and December 2015. We excluded patients < 18 years or above > 65 years and those with missing data.

Patient characteristics and risk factors

Patient characteristics included in this study are the American Joint Committee on Cancer (AJCC) stages of NHL (stages I-IV), patients' race/ethnicity defined as White (non-Hispanic White), Hispanic, Black (non-Hispanic Black) Black, and others. In addition, we stratified insurance status as private insurance, public insurance, or uninsured. The treatments include surgery, chemotherapy, and radiotherapy.

Definition of study outcome

Our primary outcome of interest is the patients' survival following diagnosis of NHL.

Statistical analysis

Descriptive statistics, including frequencies and percentages, were used to describe the baseline characteristics of patients and risk factor variables.

We utilized the Cox regression model to explore the interaction between race and insurance type and how this influences survival among NHL patients. We calculated the hazard ratio (HR) or time to death, controlling for patients' age, sex, race/ethnicity, insurance, treatment modalities (surgery, chemotherapy, or radiation), and disease stage. A two-tailed p-value <0.05 was considered statistically significant. We utilized STATA version 16 (StataCorp College Station, TX) to perform the analysis.

## Results

A total of 44,609 patients with NHL were identified from the SEER Registry that fulfilled the study criteria. Table 1 is a baseline distribution of patients' demographic characteristics and treatment modalities. The mean age was 50.9±10.8 years, with a mean survival of 49.8±34.5 months. The majority of patients were Whites 64.8%, 16.0% were Hispanics, 10.8% were Blacks, and 7.3% were Asians/Pacific Islanders. The patients identified were at various stages of NHL. Most of these patients were in disease stage IV, representing 37.0%. The conventional modality of treatment was chemotherapy at 69.5%.

**Table 1:** Baseline frequency distribution of studied variables

Variables	Frequency	Percentages
Age in Years (mean ± standard deviation)	50.9± 10.8	
Survival in Months (mean ± standard deviation)	49.8± 34.5	
Female	18,490	41.45
Male	26,119	58.55
Disease Stage at Presentation		
Stage I	12,150	27.24
Stage II	7,760	17.4
Stage III	8,181	18.34
Stage IV	16,518	37.03
White	28,893	64.77
Black	4,824	10.81
Hispanics	7,135	15.99
Asians	3,239	7.26
Others	518	1.16
Private Insurance	34,126	76.5
Public Insurance	7,420	16.63
Uninsured	3,063	6.87
Chemotherapy	31,003	69.5
Radiation	7,870	17.78
Surgery	12,374	27.74
Mortality	8,187	18.35

Table 2 is a bivariate analysis showing the distribution of patient characteristics by race/ethnicity. While whites were older (mean age: 51.97±10.24 years) and had the highest survival (52.74±34.32 months), black patients were the youngest population (mean age: 48.38±11.43 years) and had the lowest mean survival (42.92±34.37 months).

**Table 2:** Bivariate comparisons of demographic and clinical variables by race/ethnicity (SEER 2007-2015) p-value: probability-value; based on separate chi-square tests for categorical variables, student’s t-test for age and survival as a normally distributed continuous variable

Variables	White	Black	Hispanics	Asians	Others	p-value (a)
	(n=28,893)	(n=4,824)	(n=7,135)	(n=3,239)	(n=518)	
Age in years (mean ± standard deviation)	51.97±10.24	48.38±11.43	48.45±11.51	49.94±11.59	50.47±11.12	<0.001
Survival in months (mean ± standard deviation)	52.74±34.32	42.92±34.37	43.64±33.85	47.74±34.23	51.18±33.39	<0.001
Female	40.73%	41.11%	43.08%	44.83%	41.31%	<0.001
Disease Stage at Presentation						
Stage I	27.32%	24.67%	26.66%	30.56%	33.59%	<0.001
Stage II	16.98%	16.36%	18.67%	19.64%	18.92%	
Stage III	18.44%	18.91%	18.85%	15.50%	18.34%	
Stage IV	37.27%	40.07%	35.82%	34.30%	29.15%	
Private Insurance	83.78%	60.16%	57.94%	77.74%	70.66%	<0.001
Public Insurance	11.26%	27.65%	30.11%	17.26%	24.52%	
Uninsured	4.97%	12.19%	11.96%	5.00%	4.83%	
Chemotherapy	68.56%	68.14%	73.12%	73.08%	62.36%	<0.001
Radiation	18.03%	15.30%	16.7%	21.69%	17.38%	<0.001
Surgery	28.13%	24.19%	27.90%	29.39%	26.06%	<0.001
Mortality	16.27%	27.99%	20.31%	19.20%	12.55%	<0.001

Table 3 shows the Cox regression we performed, controlled for patients' age, race/ethnicity, insurance type, disease stage, and treatment modality. Male patients, Blacks, and patients on public insurance had the lowest survival following a diagnosis of NHL.

**Table 3:** Cox regression model for NHL (SEER 2007-2015) HR: hazard ratio; CI: confidence interval. (b) p-value: probability-value; Omnibus chi-square p-value < 0.001; Hosmer-Lemeshow goodness-of-fit p-value = 0.36

		95% Confidence Interval		
Variables	HR	Lower CI	Upper	P-value
Age (years)	1.08	1.02	1.02	<0.001
Sex				
Female	Reference			
Male	1.41	1.341	1.48	<0.001
Stage I	Reference			
Stage II	1.13	1.03	1.23	0.01
Stage III	1.67	1.54	1.82	<0.001
Stage IV	2.43	2.26	2.62	<0.001
White	Reference			
Blacks	1.66	1.55	1.78	<0.001
Hispanics	1.16	1.09	1.24	<0.001
Asians	1.23	1.12	1.34	<0.001
Others	0.79	0.61	1.02	0.07
Private	Reference			
Public	2.11	2.00	2.24	<0.001
Uninsured	1.76	1.62	1.91	<0.001
Chemotherapy	1.86	1.74	1.98	<0.001
Radiation	1.01	0.94	1.08	0.881
Surgery	0.86	0.82	0.91	<0.001

Table 4 is an interaction term analysis to determine the interaction between race/ethnicity, patients' insurance type, and overall survival among patients with NHL. White patients with private insurance were the reference group. Patients with public insurance had the lowest survival across all races/ethnicities.

**Table 4:** Interactions between race/ethnicity and insurance type and its effects on overall survival among NHL Patients (SEER 2007-2015) † Model adjusted for age, sex, disease stage, and treatment modalities. HR: hazard ratio; CI: confidence interval. (b) p-value: probability-value; Omnibus chi-square p-value < 0.001; Hosmer-Lemeshow goodness-of-fit p-value = 0.44; X: interaction term

		95% Confidence Interval		
Variables	HR	Lower CI	Upper CI	p-value (b)
Private X White	Reference			
Public X White	2.19	2.03	2.38	<0.001
Uninsured	1.89	1.69	2.12	<0.001
Private X Black	1.67	1.53	1.83	<0.001
Public X Black	3.63	3.29	4.02	<0.001
Uninsured X Black	2.84	2.43	3.32	<0.001
Private X Hispanic	1.20	1.01	1.32	<0.001
Public X Hispanic	2.48	2.25	2.72	<0.001
Uninsured X Hispanic	1.97	1.68	2.31	<0.001
Private X Asian	1.36	1.23	1.52	<0.001
Public X Asian	2.11	1.76	2.53	<0.001
Uninsured X Asian	1.94	1.38	2.72	<0.001

## Discussion

This study found significant differences in the disease distribution and relative survival among the different racial groups in the United States. Racial differences in the epidemiology of different malignancies have been established in the literature [9-10]. NHL is a group of heterogeneous conditions made up of more than 60 subtypes, among which diffuse large B-cell lymphoma (DLBCL), follicular lymphoma (FL), and chronic lymphocytic leukemia/small lymphocytic lymphoma (CLL/SLL) are the most common subtypes [11]. This study found a higher incidence of NHL among whites (65%) compared to other races. An earlier study of lymphoma epidemiology in the United States similarly shows a higher incidence of most lymphoid neoplasms in Whites except for plasma cell and T-cell neoplasms, which are more common in Blacks. At the same time, Asians have a lower rate of CLL/SLL when compared to Whites and Blacks [10]. While no specific factor has been identified as responsible for these disparities in incidence, disparities in outcome are mainly driven by racial differences in socioeconomic, behavioral, environmental, structural, and biological factors [10]. A recent study on the effect of genetic ancestry on tumor genomic alterations in diffuse large B-cell lymphoma found similar mutations affecting PIM1, MYD88, MLL2, HIST1H1E, and BCL2 genes across European, African, and Asian ancestry groups, suggestive of an identical underlying mechanism of disease pathogenesis [12]. However, the study also found a significantly higher incidence of six driver genes (ATM, SETD2, MGA, MLL3, TET2, DNMT3A) in African ancestry compared to European ancestry [12]. Similar disparities have been reported in the incidence of other non-hematological malignancies such as breast, lung, colon, and prostate [10].

This study finds a significant association between having private insurance and a higher relative survival in NHL. While many factors, including age, tumor biology, tumor stage, and comorbidities, are important determinants of survival, access to healthcare, driven in part by the type of insurance, is an essential determinant of long-term outcome in cancer care [13]. Among our study population, three-fourths of individuals with NHL were on private insurance, with 17% on public insurance and 7% uninsured. In addition, patients with private insurance have a higher relative survival across all races/ethnicity. The association between insurance type and survival may be potentially confounded by demographic parameters such as socioeconomic factors and age. Age is an important prognostic factor in NHL. A recent study involving 763,884 patients with breast, ovarian, endometrial, cervical, colon, lung, and gastric cancer who were on public insurance or uninsured showed these patients had significantly lower odds of receiving appropriate cancer chemotherapy, radiotherapy, or surgery when compared to those on private insurance. These study findings persisted even after adjusting for demographic parameters [14].

Similarly, Niu et al. investigated 54,002 patients in New Jersey with breast, cervical, lung, colorectal, prostate, and bladder cancers, and NHL and found a higher risk of death in NHL, colorectal, breast, lung, and prostate cancer patients who are uninsured or Medicaid insured [15]. Other studies have demonstrated a lower odds of receiving guideline therapy in uninsured and those on public insurance than those on private insurance [16-17]. In addition, late presentation and late-stage disease at diagnosis are higher in the uninsured and those on public insurance leading to a poorer prognosis [18-19]. The association of insurance type and survival outcome could be due to one or a combination of many factors, including delay in seeking care early due to deterrence of out-of-pocket expenses, difficulty accessing specialists who are willing to accept insured/public insurance patients, challenges with gaining access to newer more effective and less toxic therapies, which could be more expensive and unavailable to those who are uninsured or on public insurance, and a higher likelihood of seeking care in safety-net-hospitals, which may be lacking in newer cutting edge cancer management technologies [13].

While private insurance is often considered the gold standard for optimal access to health care, 16.6% of patients in this study are on public insurance while 6.9% are uninsured. This study shows that relative survival is worse in public insurance individuals than in the uninsured. Although a lower quality of care is not unexpected in the uninsured, those on public insurance have been shown to equally receive a lower quality of care than private insurance, which might be responsible for the worse survival outcome. Our findings are similar to other studies on breast and colorectal cancers [17,20]. Roetzheim et al. found a higher mortality rate among breast cancer patients on Medicaid insurance (RR, 1.58, 95% CI, 1.18-2.11; P = 0.002) and the uninsured (R.R., 1.31; 95% CI, 1.03-1.68; P = 0.03) when both were compared to patients who utilized private insurance [17]. A similar study on colorectal cancer found a higher relative risk of death in Medicaid recipients than in the uninsured [20]. However, another study on breast cancer reported a worse survival outcome in the uninsured than in patients with public insurance [21]. In addition, a study on survival outcomes in breast, lung, colorectal, and prostate cancers found no difference in survival outcomes between the two groups [22]. This inconsistent pattern suggests a complex relationship between relative survival and being uninsured or having public insurance coverage, with many factors playing a role.

This study finds that black patients have the worst relative survival compared to other groups with NHL. However, due to the heterogeneous nature of NHL, survival among the various subtypes may vary. In a study of the three most common subtypes of NHL (DLBCL, FL, and CLL/SLL), Li et al. found a significantly higher five-year relative survival among Whites with stages I-IV DLBCL compared to Blacks, Asians, and Pacific Islanders, while Blacks have the worst five-year relative survival among patients with stages I-III, and Asians/Pacific Islanders have the worst survival in stage IV [11]. However, the study did not show a consistent pattern of racial disparity across disease stages in FL and CLL/SLL. Our study found Blacks have the worst survival even after adjusting for age, sex, disease stage, insurance type, and treatment modalities, suggesting a complex interplay of many factors.

The study was limited to people between 18 and 65 years. These excluded many NHL patients and may significantly impact the results. Second, the SEER database lacks specific information on some risk factors like comorbidities, body mass index (BMI), hormonal exposure, human papillomavirus (HPV) status, and details on smoking habits. There is also a lack of data in the database about specific chemotherapy, treatment sequence, radiation doses, and modifications in treatment regimens.

## Conclusions

The present study reinforces the previously established notion of significant racial disparity in outcomes following NHL. White patients and those with private insurance continue to have the best outcomes. Though the disparity in cancer survival is multifactorial, tumor biology and the interaction between patients' race/ethnicity and insurance type continue to play a significant role in outcomes of NHL in the United States. While the elimination of health disparities among all sociodemographic groups remains an area of interest, it is equally important to focus future research on understanding the role of tumor genetics in the racial disparities seen in many cancers including NHL.

## References

[1] Sung H, Ferlay J, Siegel RL, Laversanne M, Soerjomataram I, Jemal A, Bray F (2021). Global Cancer Statistics 2020: GLOBOCAN estimates of incidence and mortality worldwide for 36 cancers in 185 countries. CA Cancer J Clin.

[2] Mukhtar F, Boffetta P, Dabo B (2018). Disparities by race, age, and sex in the improvement of survival for lymphoma: findings from a population-based study. PLoS One.

[3] Thandra KC, Barsouk A, Saginala K, Padala SA, Barsouk A, Rawla P (2021). Epidemiology of non-Hodgkin's lymphoma. Med Sci (Basel).

[4] de Leval L, Jaffe ES (2020). Lymphoma classification. Cancer J.

[5] Sapkota S, Shaikh H (2021). Non-Hodgkin Lymphoma. https://www.ncbi.nlm.nih.gov/books/NBK559328/.

[6] Howlader N, Noone AM, Krapcho M (2019). SEER Cancer Statistics Review 1975-2016. SEER Cancer Statistics Review.

[7] Shavers VL, Brown ML (2002). Racial and ethnic disparities in the receipt of cancer treatment. J Natl Cancer Inst.

[8] Tao L, Foran JM, Clarke CA, Gomez SL, Keegan TH (2014). Socioeconomic disparities in mortality after diffuse large B-cell lymphoma in the modern treatment era. Blood.

[9] Morton LM, Wang SS, Devesa SS, Hartge P, Weisenburger DD, Linet MS (2006). Lymphoma incidence patterns by WHO subtype in the United States, 1992-2001. Blood.

[10] Zavala VA, Bracci PM, Carethers JM (2021). Cancer health disparities in racial/ethnic minorities in the United States. Br J Cancer.

[11] Li Y, Wang Y, Wang Z, Yi D, Ma S (2015). Racial differences in three major NHL subtypes: descriptive epidemiology. Cancer Epidemiol.

[12] Lee MJ, Koff JL, Switchenko JM (2020). Genome-defined African ancestry is associated with distinct mutations and worse survival in patients with diffuse large B-cell lymphoma. Cancer.

[13] Marlow NM (2009). The Relationship Between Insurance Coverage and Cancer Care: A Literature Synthesis.

[14] Parikh-Patel A, Morris CR, Kizer KW (2017). Disparities in quality of cancer care. The role of health insurance and population demographics. Medicine (Baltimore).

[15] Niu X, Roche LM, Pawlish KS, Henry KA (2013). Cancer survival disparities by health insurance status. Cancer Med.

[16] Mitchell JM, Meehan KR, Kong J, Schulman KA (1997). Access to bone marrow transplantation for leukemia and lymphoma: the role of sociodemographic factors. J Clin Oncol.

[17] Roetzheim RG, Gonzalez EC, Ferrante JM, Pal N, Van Durme DJ, Krischer JP (2000). Effects of health insurance and race on breast carcinoma treatments and outcomes. Cancer.

[18] Roetzheim RG, Pal N, Gonzalez EC, Ferrante JM, Van Durme DJ, Krischer JP (2000). Effects of health insurance and race on colorectal cancer treatments and outcomes. Am J Public Health.

[19] Halpern MT (2008). Association of insurance status and ethnicity with cancer stage at diagnosis for 12 cancer sites: a retrospective analysis. Lancet Oncol.

[20] Chen AY, Schrag NM, Halpern MT, Ward EM (2007). The impact of health insurance status on stage at diagnosis of oropharyngeal cancer. Cancer.

[21] Bradley CJ, Given CW, Roberts C (2003). Correlates of late stage breast cancer and death in a Medicaid-insured population. J Health Care Poor Underserved.

[22] Ward E, Halpern M, Schrag N (2008). Association of insurance with cancer care utilization and outcomes. CA Cancer J Clin.

